# Targeting Subviral Particles: A Critical Step in Achieving HBV Functional Cure but Where Are We with Current Agents in Clinical Development?

**DOI:** 10.3390/v14061193

**Published:** 2022-05-31

**Authors:** Andrew Vaillant

**Affiliations:** Replicor Inc., 6100 Royalmount Avenue, Montreal, QC H4P 2R2, Canada; availlant@replicor.com; Tel.: +1-514-733-1998

The recent review [1] by Drs. Moini and Fung on HBsAg loss as an important treatment endpoint for functional cure provides a good overview of the different compounds in development for the treatment of chronic HBV. However, some clarifications are warranted based on the evolution of clinical data in the field with a variety of investigational agents over recent months.

ALG-010133 is a nucleic acid polymer (NAP) that was under development by Aligos Therapeutics. This NAP is a locked nucleic acid (LNA)-modified version of the NAP REP 2165 [2] previously developed by Replicor Inc. The LNA modification was abandoned more than a decade ago during the development of nucleic acid polymers (NAPs) at Replicor. Predictably, ALG-010133 monotherapy is accompanied by significant injection site reactions [3] and has no activity in HBV infected patients at the 400 mg dose [4], whereas active NAPs (REP 2139 and REP 2055) achieve up to 7 log_10_ IU/mL HBsAg reduction from baseline with monotherapy at similar doses and dosing duration [5]. The development of all LNA-modified NAPs has been halted [4]. The mechanistic reasons for the failure for ALG-010133 and why the LNA modification was discarded early in the development of NAPs have been recently detailed [2]. Development of the HBV LNA antisense oligonucleotide (ASO) ALG-020572 has also been canceled due to hepatoxicity [6], an expected complication of the LNA modification that was employed in this ASO [2].

A recent study demonstrating the ability of cccDNA to establish and maintain a stable cccDNA copy number in the absence of the core antigen [7] draws into question the potential clinical impact of capsid assembly modulators (CAMs) in chronic HBV infection. Several class I and class II capsid assembly modulators (CAMs) have been evaluated in monotherapy. As expected, all have elicited strong declines in HBV DNA and HBV RNA in agreement with their mechanism of action; however, none have shown any effects on HBsAg in the absence of nucleos(t)ide analogs (NUCs). Moreover, the lack of effect of CAM monotherapy on cccDNA activity (HBeAg and HBcrAg), despite the rapid turnover of active cccDNA [8] is consistent with the lack of requirement of HBcAg for cccDNA maintenance that was previously described [7]. On the other hand, all NUCs, either approved or in development (ETV, TDF, TAF, or the protide version of clevudine [ATI-2173]), have well-described secondary effects in stimulating innate immunity [9,10,11,12,13,14,15,16]. Moreover, in the clinic, NUC monotherapy is accompanied by mild declines in HBsAg and stronger declines and/or clearance of HBeAg, HBV RNA and HBcrAg [17,18,19,20,21]. As such, the universal rebound of infection following long term combination therapy with NUCs and vebicorvir [22] and the subsequent halt of its development draws into question the contribution that CAMs can make in the pursuit of a functional cure. The field is anticipating results with more potent CAMs in development.

It should be noted that nucleic acid polymers (NAPs) are accompanied by a reduction in HBsAg that is far greater than the typical 2–3 log_10_ IU/mL reduction from baseline that was observed with RNAi when combined with NUCs and CAMs [23]. NAPs achieve HBsAg reductions of up to 8 log_10_ IU/mL from baseline [24] with 70% of patients achieving HBsAg < 1 IU/mL, 60% of whom achieve HBsAg loss (<0.005 IU/mL) [21,24]. These effects of NAPs on circulating HBsAg are attributed to their ability to selectively target the assembly and secretion of HBV subviral particles (SVP) [25] which constitute >99.99% of circulating HBsAg [26]. This antiviral effect was recently validated in clinical studies where the small isoform of HBsAg was selectively cleared during therapy with NAPs, which is diagnostic of targeting of SVPs [27]. Importantly, NAPs have no effect on the production and or release of Dane particles or HBeAg [25].

Contrary to what is stated by Moini et al. [1], quantitative HBsAg is not well correlated with intrahepatic cccDNA [28,29,30,31] even in HBeAg positive patients [29], due to the contribution of SVP to the HBsAg pool. SVPs are produced from both active cccDNA and integrated HBV DNA [32]; integration is an early and cumulative event over the natural progression of chronic HBV infection. HBV DNA integration is present in HBeAg positive infection [33], albeit at lower levels than in HBeAg negative infection.

The targeting of HBsAg loss must include the efficient removal of SVP. In this regard, several fundamental misconceptions still exist regarding the mechanisms by which HBsAg reduction is achieved during therapy with RNAi or ASO compounds that are designed to target the degradation of HBV mRNA. HBV infection has a very high degree of genetic plasticity (thousands of quasispecies exist in any particular patient) [34], a result of the lack of proofreading activity of the HBV reverse transcriptase, the rapid turnover of cccDNA and the constant immune pressure that is exerted on the virus. Given that single point mutations abolish the ability of RNAi or ASOs to target specific mRNA cleavage [35], the theoretical basis for the consideration of RNAi or ASO approaches is not clear. This was borne out by the failure of ARB-1467 (TKM-HBV), an LNP formulated RNAi with three siRNA triggers: two in HBsAg and one in HBx (thus covering cccDNA and integrated HBV DNA). LNP formulation of siRNA is the most efficient delivery method for RNAi compounds, resulting in the functional uptake of RNAi compounds, exclusively in hepatocytes [36,37]. In NUC-suppressed chronic HBV infection, ARB-1467 had minimal effects on HBsAg and, importantly, initial mild declines in HBV RNA and HBcrAg rapidly rebounded during treatment [38]. Additionally, a recent HBsAg isoform analysis (using the same assay platform that was used to evaluate NAPs) during therapy with AB-729 and a GalNAc-conjugated single RNAi trigger in HBx revealed no targeting of SVP, but rather the reverse, selective targeting of virions [39]. Both observations exclude mRNA degradation as a potential mechanism for HBsAg reduction by compounds designed as RNAi or ASO for chronic HBV infection.

What then is the mechanism underlying the HBsAg reductions that were observed with RNAi and the lone remaining HBV ASO compound in development (bepirovirsen)? All oligonucleotides have off-target immunoreactive properties. In the case of single stranded DNA, it is the stimulation of the innate immune response via TRL9 by CpG motifs [40]. Bepirovirsen (GSK 3228836) has a cryptic class II CpG motif in its 5′ end [26]. Unconjugated bepirovirsen accumulates mainly in Kupffer cells (as is the case for all phosphorothioate oligonucleotides), while GalNAc-conjugated bepirovirsen (GSK3389404) accumulates primarily in hepatocytes [41]. Unconjugated bepirovirsen therapy in chronic HBV infection leads to strong HBsAg reduction but only in patients with low HBsAg (<1000 IU/mL) at baseline, an observation that is inconsistent with an antisense effect [42]. This HBsAg decline was accompanied by the strong activation of immune responses consistent with the stimulation of TLR9 [43]. The GalNAc-conjugated variant of bepirovirsen (GSK 3389404) has none of these activities in chronic HBV infection [44], again excluding an antisense effect.

For any RNAi, stimulation of TLR3 is a foregone conclusion: the stimulation of TLR3 occurs with any double-stranded RNA (dsRNA) and is sequence independent [40]. It is possible to quench the TLR reactivity of dsRNA with extensive modification but this blocks the RISC-loading that is required for an RNAi effect [45]. RISC-mediated mRNA cleavage with RNAi reliably elicits a rapid effect on protein levels with a variety of liver proteins, yielding a 1 log_10_ reduction in protein levels within 15 days following the first RNAi dose. For all GalNAc-RNA evaluated to date in chronic HBV infection, many subjects did not experience any significant or reductions fare weaker than a 1 log_10_ reduction in HBsAg from baseline for 4 weeks after the first dose. Given the rapid turnover rate of SVP with a ½ life of 1–6 days [46,47], the absence of a 1 log_10_ decline in HBsAg two weeks after the first administered dose of all GalNAc-RNAi (in addition to the HBsAg isoform response analysis during AB-729 treatment mentioned above) strongly indicates that an RNAi effect is absent in these patients.

In switching from LNP formulation to GalNAc conjugation for RNAi delivery, the functional accumulation of RNAi (relative to LNP) in Kupffer cells becomes substantially higher, allowing for enhanced stimulation of TLR3. For all GalNAc-RNAi evaluated to date in chronic HBV infection, a delayed response in HBsAg occurs universally one month after the first dose of GalNAc-RNAi, gradually becoming saturated at 1.5–2 log_10_ reduction from baseline. This HBsAg response is remarkably similar to the delayed HBsAg response to TLR3 stimulation by poly I:C in the HDI HBV mouse model [48]. All of the current HBsAg response data with ASOs and LNP- or GalNAc-RNAi in human trials are inconsistent with effects on HBsAg decline expected from the degradation of mRNA; however, HBsAg responses (as well as markers of cccDNA activity) are entirely consistent with the inactivation/degradation of cccDNA-mediated by TLR9/TLR3-stimulated innate immune responses from these compounds.

A critical milestone in the achievement of functional cure is the clearance of HBsAg, which in turn requires elimination of SVP from the blood. Understanding the mechanisms, advantages and limitations of various agents is critical to designing optimal combination regimens which can achieve high rates of functional cure with finite therapy.
